# Molecular-Scale Dynamics of Long Range Retrograde Brain-Derived Neurotrophic Factor Transport Shaped by Cellular Spatial Context

**DOI:** 10.3389/fnins.2022.835815

**Published:** 2022-03-31

**Authors:** Anke Vermehren-Schmaedick, Mark J. Olah, Damien Ramunno-Johnson, Keith A. Lidke, Michael S. Cohen, Tania Q. Vu

**Affiliations:** ^1^Department of Biomedical Engineering, Knight Cancer Institute, Oregon Health and Science University, Portland, OR, United States; ^2^Department of Chemical Physiology and Biochemistry, Oregon Health and Science University, Portland, OR, United States; ^3^Department of Physics and Astronomy, University of New Mexico, Albuquerque, NM, United States

**Keywords:** BDNF, quantum dot, retrograde transport, single particle tracking, molecular dynamics, DRG, subcellular architecture, axon-dendrite

## Abstract

Retrograde neurotrophin (NT) transport is a specialized form of signal transduction used to conduct information from axons to the cell bodies of central and peripheral nervous system neurons. It is activated upon NT-Trk receptor binding, NT-Trk internalization into signaling endosomes, and their motion along the axon toward the cell body. Brain-derived neurotrophic factor (BDNF) is an abundant NT that modulates key brain and spinal cord functions, and defects in BDNF trafficking are associated with neuronal death, neurodegenerative diseases and in nerve injury. Decades of study have yielded impressive progress in elucidating NT retrograde transport; however, much information remains unclear. For example, while it is known that NT function is dependent on tight control of NT-receptor intracellular trafficking, data describing the precise spatiotemporal molecular dynamics of their axonal to somatic transport are lacking. In past work, we showed the use of discrete, photo-bleaching-resistant quantum dot (QD)-BNDF probes to activate and track BDNF-TrkB receptor internalization; this revealed a rich diversity of molecular motions that intracellular BDNF signaling endosomes undergo within the soma of nodose ganglia sensory neurons. Here, we used combined techniques of discrete QD-BDNF tracking with compartmented microfluidic chambers to characterize retrograde BDNF-TrkB transport over long-ranging distances of primary dorsal root ganglion sensory neuronal axons. Our new findings show that axonal retrograde motion is comprised of heterogeneous mixtures of diffusive behaviors, pauses, and variations in net molecular-motor-dependent transport speeds. Notably, specific molecular dynamic features such as NT speed were dependent on spatial context that could be categorized in distance from distal axons and proximity to the soma and were not entirely dictated by active motor transport speed. The important implication is recognition that NT-receptor retrograde transport is comprised of molecular dynamics, which change over the course of long-range trafficking to shape overall transport and possibly signaling.

## Introduction

Neurons are highly polarized cells with extensive dendritic arborization and elongated axons that can span over a meter from their cell bodies. To efficiently conduct signal transduction over such long axonal distances, neurons use the retrograde endosomal trafficking as a mechanism of intracellular signaling. Neurotrophin (NT) ligands are found in abundance in diverse neuronal systems (e.g., central nervous system, sensory systems, and spinal cord) and play an important role in signaling basic neural functions. Several decades of work reveal that NT signaling employs retrograde endosomal signaling that involves initial binding of the NT to a receptor tyrosine kinase at distal axons, followed by internalization and net movement of the NT-receptor complex in signaling endosomes toward the neural cell body. Furthermore, mutations in some of the proteins involved in NT trafficking can cause affect transport or cause a complete block in axonal transport (Sleigh et al., 2019; Guillaud et al., 2020). Brain-derived neurotrophic factor (BDNF) is a member of the NT family of neuronal growth factors that are produced and secreted by non-neuronal tissues as well as neurons in the peripheral and central nervous systems. During neurodevelopment, BDNF promotes neuronal survival, differentiation and neurite outgrowth in sympathetic and sensory neurons (Huang and Reichardt, 2001, 2003; Ascano et al., 2012; Yap and Winckler, 2012; Park and Poo, 2013; Bronfman et al., 2014; von Bartheld, 2014), and in response to axonal injury, BDNF promotes regeneration of injured axons (Tonra et al., 1998; Michael et al., 1999; Zhou et al., 1999; Deng et al., 2000; Song et al., 2008; Geremia et al., 2010). Like other NTs, BDNF signaling is conveyed to the cell body via signaling endosomes that contain BDNF-Trk receptors that are retrogradely transported axon-to-nucleus (Watson et al., 1999; Zweifel et al., 2005; Chowdary et al., 2012; Schmieg et al., 2014; Olenick and Holzbaur, 2019). Because disruptions in retrograde transport of NT underlie the etiology of several neurological diseases, including Alzheimer’s, Huntington’s, Parkinson’s, and amyotrophic lateral sclerosis (Zweifel et al., 2005; Millecamps and Julien, 2013; Neefjes and van der Kant, 2014), a complete understanding of retrograde NT signaling transport is high in importance.

The macroscopic dynamics of retrograde NT transport have been long studied but now a focus on their molecular-resolved dynamics is possible due to technical progress in the use of bright, photostable quantum dots (QDs) to perform single molecule tracking and the use of compartmented microfluidic devices to provide spatial separation of neural axons (Segal, 2003; Zhang et al., 2010; Chowdary et al., 2012; Neto et al., 2016). QDs are fluorescent nanoparticles that exhibit high photostability and quantum efficiency (Chang et al., 2008; Vu et al., 2015) in comparison to other commonly used fluorescent dyes (e.g., Cy3, GFP, and RFP) (Poon et al., 2011; Zhou et al., 2012; Bronfman et al., 2014; Matusica and Coulson, 2014). The properties allow continuous tracking of NTs, over several minutes, at a high frame rate, allowing observation of both long- and short-timescale processes. Yet, while the use of QDs for single molecule tracking studies have started to uncover more detailed understanding of transport of NT signaling endosomes, such single molecule studies remain limited in number and have largely focused on details of active transport (e.g., microtubule motor-directed motion) (Howe and Mobley, 2003; Zhang et al., 2010; Xie et al., 2012; Harrington and Ginty, 2013; Bronfman et al., 2014; Cosker and Segal, 2014; Zhao et al., 2014; De Nadai et al., 2016).

Early evidence suggests that NT transport may span a rich complexity of molecular motions that is beyond active transport. In hippocampal neurons, NGF endosomes exhibit diffusive and pauses in active motion that are attributed to local structures within the axon (Chowdary et al., 2012). In addition, we have found, by using discrete QD-labeled BDNF (BDNF-QD) probes to evoke BDNF-Trk signaling, that the intracellular motion of endocytosed BDNF endosomes in the cell bodies of nodose ganglia sensory neurons also showed heterogeneous dynamics; with active transport present following curvilinear trajectories (Vermehren-Schmaedick et al., 2014, 2019). Here, we sought to characterize the molecular dynamic behavior of BDNF-TrkB endosomes in retrograde transport along axons: at their distal tips, along the axonal shaft, and proximal to the neuronal cell body in dorsal root ganglion (DRG) sensory neurons. DRG neurons have extensive axons that span hundreds of microns in length and they express the BDNF receptor TrkB (Foster et al., 1994) which, upon BDNF stimulation, undergoes retrograde transport of the BDNF-TrkB complex that underlies neuronal survival signaling (Heerssen et al., 2004). We used single-molecule high-resolution imaging of BDNF-QD and microfluidic devices to conduct the first extensive study examining the molecular motion of BDNF-NT transport and its dependence on a subcellular spatial context along different portions of the axons.

## Materials and Methods

### Ethics Statement

All procedures were approved by the Institutional Animal Care and Use Committee of the Oregon Health and Science University (Protocol Number: IS00001990), and conformed to the Policies on the Use of Animals and Humans in Neuroscience Research approved by the Society for Neuroscience and the NIH Guide for the Health and Use of Laboratory Animals.

### Microfluidic Devices for Compartmentalization of Axons and Cell Bodies

The preparation of the microfluidic devices was done as described in Cohen et al. (2011), with the following modifications: the microfluidic culturing devices had 160 microgrooves of 450 μm × 10 μm, and were made in the lab using polydimethylsiloxane (Sylgard 184; Dow Corning, Midland, MI, United States). Glass bottom culture dishes (40 mm diameter; Ted Pella, Redding, CA, United States) were coated with 0.01% Poly-D-Lysine (PDL; Trevigen, Gaithersburg, MD, United States) for 12 h at 37°C, in a tissue culture (TC) incubator containing 5% CO_2_. After washing and drying the dishes, the sterile microfluidic devices were reversibly affixed to the PDL coated glass, and the four reservoirs of the devices were incubated with 5 μg/ml mouse laminin I (Trevigen, Gaithersburg, MD, United States) in NeuroQ (NQ) basal medium (GlobalStem, Rockville, MD, United States) for at least 3 h at 37°C in a TC incubator. Laminin I was then exchanged for NQ medium and the dishes were kept at 37°C until neurons were added. Dishes with the devices were placed in square petri dishes with sterile water to reduce evaporation rate of media.

### Preparation of Dorsal Root Ganglion Cultures

Embryos (E14) were obtained from time pregnant Sprague Dawley rats (Charles River Laboratories, Wilmington, MA, United States). Pregnant female rats were euthanized by exposure to CO_2_. Embryos were excised in HBSS buffer (Gibco, Life Technologies, Carlsbad, CA, United States), DRGs were removed and collected in ice-cold HBSS buffer, digested in 0.25% trypsin (Gibco, Life Technologies, Carlsbad, CA, United States) and 1 mg/ml DNAseI (Roche, Indianapolis, IN, United States) for 15 min at 37°C, followed by dissociation in plating medium [PM: NQ medium supplemented 10% fetal bovine serum (HyClone, Logan, UT, United States), 1% GlutaMAX, 1% sodium pyruvate, 1% Penicillin-Streptomycin-Neomycin antibiotic mixture (Life Technologies, Carlsbad, CA, United States)]. DRG neurons were centrifuged for 3 min at 800 *g* and resuspended in 200 μl culture medium [CM: NQ medium supplemented with GS21 (GlobalStem, Rockville, MD, United States), GlutaMAX, 1% Penicillin-Streptomycin-Neomycin antibiotic mixture (Life Technologies, Carlsbad, CA, United States)] and counted. Dissociated neurons were plated (5 μl) in the cell body compartment of the microfluidic devices at 1.5 × 107 cells/ml, and let adhere to the glass. Following a 30 min incubation at 37°C, 150 μl of CM supplemented with 50 ng/ml NGF (PeproTech, Rocky Hill, NJ, United States; Cat# 450-01) and 1× 5-Fluorodeoxyuridine (FdU; MP Biomedicals, Santa Ana, CA, United States) [CM++] were added to each microfluidic well. CM+ was replaced every 2 days until treatment.

### Generation of Brain-Derived Neurotrophic Factor-Quantum Dot Probes

Quantum-dot BDNF (BDNF-QD) probes were prepared as described in Vermehren-Schmaedick et al. (2014). Briefly, probes were produced by high-affinity binding of biotinylated BDNF to streptavidin-QDs. Biotinylated BDNF was obtained by covalent linkage of biotin to the lysine and N-terminal amino groups of recombinant human BDNF (Pepro-Tech; Cat# 450-02). BDNF-QD probes were made fresh by incubating biotinylated BDNF with streptavidin-QD655 (Life Technologies, Carlsbad, CA, United States; Cat# Q10121MP) at a molar ratio of 1:1. The vast majority of QD signals that we tracked moving within the axons were single BDNF-QDs. This is based on the facts that each one of them displayed “blinking,” a characteristic of single QDs, and on our previous validation of the BDNF-QD probes (Vermehren-Schmaedick et al., 2014).

### Brain-Derived Neurotrophic Factor-Quantum Dot Stimulation of Neuronal Endings

**FIGURE 1:**
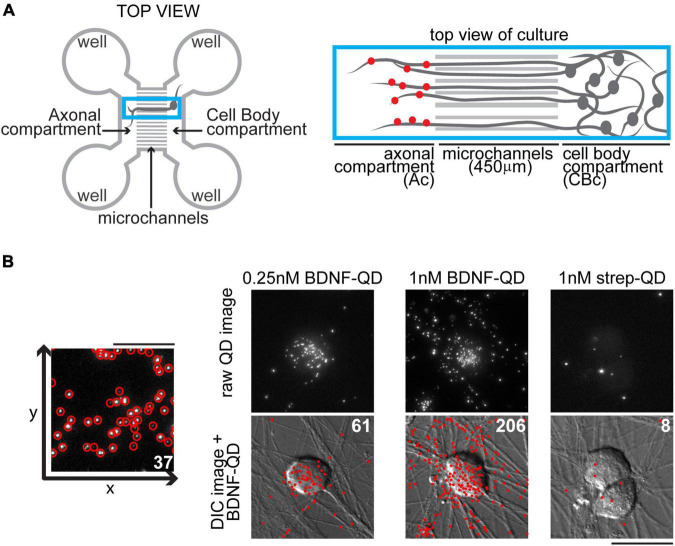
Compartmentalization of dorsal root ganglion (DRG) cultures in microfluidic devices and discrete brain-derived neurotrophic factor-quantum dot (BDNF-QD) probe localization and quantification. **(A)** Representation of a microfluidic device designed to structurally and fluidically isolate axons from cell bodies (left) showing the axonal and cell body compartments and microchannels. The blue box inset (right) shows a close up of the axonal and cell body compartments (Ac and CBc) connected via the microchannels. To induce retrograde transport, BDNF-QDs (red dots) were added to the Ac, and visualized in the microchannels and CBc at later time points. **(B)** Our automated software detects and quantifies single BDNF-QDs molecules with high sensitivity. Left: A schematic of a raw QD image showing the program’s identified QDs (red circles), scale bar 10 μm. Right: A comparison of three experimental conditions, BDNF-QDs (0.25 and 1 nM) and strep-QD (1 nM streptavidin-QD, control), added directly to the neurons and showing accurate identification and counting of QDs. Top images represent raw QD images (max projection of 20 slices, 5 μm total, equivalent of one third of the cell height); bottom images represent DIC images overlaid with BDNF-QD (red dots generated by Matlab obtained from the from raw QD images). The total numbers of QDs are indicated in white (top right), scale bar 25 μm.

To fine-tune our QD identification/counting software, neuronal cultures (5 days *in vitro*) grown on cover slips were pre-incubated in culture medium without GS21 supplement for 30 min at 37°C, incubated for 5 min with BDNF-QD probes (250 pM or 1 nM) or control streptavidin-QDs (1 nM), rinsed in PBS, fixed in 4% PFA at room temperature, rinsed in PBS, and placed in borate buffer (10 mM, pH 8.0) for immediate imaging (see “Imaging of BDNF-QD probes in fixed neurons and analysis,” and Figure 1). For BDNF-QD trafficking using microfluidic devices and imaged after fixing, axonal chambers were pre-incubated as above, followed by 250 pM BDNF-QD (15 or 60 min) or 250 pM control QD (60 min). To block active transport, EHNA hydrochloride (Sigma, St. Louis, MO, United States) was used at a 1 μM final concentration and added to axonal and cell body compartments 1 h prior adding 250 pM BDNF-QD). All incubations were done at 37°C, washed in PBS three times for 3 min each, fixed and placed in borate buffer for immediate imaging (only microchannels in the center of the array were imaged to avoid edge variability). To label neurons with endings in the axonal compartment, we added Vybrant CM-DiI Cell-Labeling Solution (Life Technologies, Carlsbad, CA, United States) at 1:1,000 dilution to the axonal endings 1 h prior adding BDNF-QD. For BDNF-QD trafficking and imaging in live cells, neurons (axonal and cell body chambers) were quickly rinsed with PBS, and incubated for 30 min at 37°C with FluoroBrite DMEM (Life Technologies, Carlsbad, CA, United States) medium supplemented with 50 ng/ml Vitamin C (ascorbic acid; Sigma, St. Louis, MO, United States), 25 mM HEPES and 1% GlutaMAX (Life Technologies, Carlsbad, CA, United States). Media in the axonal compartments was replaced with 250 pM BDNF-QD655 for 10 min, rinsed, kept at 37°C on a warmed microscope stage (see “Imaging of BDNF-QD probes in live neurons”) and imaged within the next 2 h (*t* = 20, 60, and 120 min). To eliminate the possibility of free diffusion of BDNF-QDs across the microchannels, we ensured that the volume in the axonal compartment was always 100 μl less than in the cell body compartment (200 μl for axonal well, 300 μl for cell body well). Streptavidin-QDs were used as negative controls.

### Imaging and Analysis of Brain-Derived Neurotrophic Factor-Quantum Dot Probes in Fixed Neurons

Neurons were imaged with an inverted epifluorescent Zeiss Observer microscope (40x/1.4 oil objective), equipped with a MLS203P2 x–y stage (ThorLabs, Sterling, VA, United States) and an Andor Luca EMCCD camera (Andor Technology, South Windsor, CT, United States). Stage and image acquisition was done using Micro-Manager software (v1.4.13). QD fluorescence was detected using filter cubes for QD655 (Excitation 434 nm, Emission 655 nm; Semrock, Rochester, NY, United States). In all experiments, cells were imaged over the total height of the cells by acquiring z-stacks (z-step = 250 nm). A lab-built MicroManager plugin was used to image the cells. One pixel represents 195 nm. Raw images were corrected for uneven illumination by applying a flat field correction (shading correction), in where each image was divided by its reference image (same exposure and optics but without a sample) using a lab-built Matlab program. The Grid/Collection Stitching Fiji (“Fiji Is Just ImageJ”)^1^ plugin (Preibisch et al., 2009) was used to perform the stitching of the multiple fields of view. Using customized Matlab software (Mathworks, Natick, MA, United States) developed in our laboratory, discrete QD fluorescence was detected, localized and tabulated. Briefly, detection of discrete QD probes was accomplished in single-cell Max Projections by applying a spatial bandpass filter, detecting localized maxima, and calculating the position of each QD for image. The output of these automated algorithms was total counts of detected and localized discrete QD fluorescent puncta in each image. The QDs were identified as discrete, non-aggregated units comprised of single or a few QDs, as confirmed by intensity profile measurements of QD fluorescence. Our automated identification and counting of QDs was accurate, with an error of less than 5% when compared to manual QD identification and count (average of 17.8 and 18.8, respectively, *n* = 10 images; and average of 116 and 121, respectively, *n* = 10 images).

### Imaging of Brain-Derived Neurotrophic Factor-Quantum Dot Probes in Live Neurons

Live neuronal imaging was performed using an inverted epifluorescent microscope and camera (Zeiss Axiovert 200M, PlanAPO 63x/1.4 oil objective, Andor iXon Ultra 897 EMCCD camera). Glass bottom culture dishes (Ted Pella, Redding, CA, United States) with DRG neurons were maintained at a constant temperature of 37°C during live imaging using a heating inset (model P S1), a heating ring for the 63x objective, and a temperature control unit (Temp Module S1, PeCon GmbH, Germany). QD fluorescence was detected using filter cubes for QD655 (Excitation 434 nm, Emission 655 nm; Semrock). Neutral density filters (1.6) were used to avoid low wavelength damage to cells. Prior to imaging, signal-to-noise ratios we checked for tracking adequacy (gain 0.12–0.14, read noise 0.6–0.65). Following BDNF-QD stimulation (250 pM pulses), movies were acquired at a fixed z plane, with ROIs of 512 × 512, and a frame rate of 17 f/s (2,000 frames in 120 s). One pixel represents 127 nm.

### Single Particle Tracking Analysis of Brain-Derived Neurotrophic Factor-Quantum Dots

Image sequences captured with the EMCCD camera were gain-calibrated to convert the native ADU units of the camera sensor to photon counts by subtracting an experimentally measured camera offset and multiplying by a gain factor determined from static calibration images (Lidke et al., 2004, 2005). The gain corrected images were then filtered using a difference-of-Gaussians spot enhancing filter to identify image regions containing likely single emitter spots (Jaqaman et al., 2008). These candidate spots were fit using a maximum-likelihood based parameter estimation procedure to estimate discrete BDNF-QD locations to sub-pixel precision (Smith et al., 2010). Filtering based on estimated spot intensity and quality of the fit was used to select only the most likely single emitter locations. These candidate emitters locations were identified for each frame and connected into trajectories using a modified version of the cost-matrix approach (Jaqaman et al., 2008) that accounts for the birth and death probabilities specific to QD blinking. All resulting trajectories were then analyzed using several common statistical measures typically used in single particle tracking analysis, such as net displacement, net velocity, and trajectory duration (Meijering et al., 2012). Next, individual trajectories of interest were identified using these track statistics combined with 3D visualization showing trajectories in dimensions of X, Y, and T, over a background image that shows the time-averaged static image obtained by blending the max-image with the sum-image (examples in Supporting Information). These selected trajectories of interest where then further analyzed by segmentation into shorter sub-trajectories, where each sub-trajectory encompassed a particular type of motion. In particular, for the axonal transport of BDNF-QDs that displayed the typical molecular motor profile of sustained liner motion interspersed with pauses, the segments allowed us to separately characterize the statistics of the motion during pauses and active transports events. All analysis was done using custom MATLAB software.

### Diffusion Constant Estimation

Diffusion constants were estimated using a maximum likelihood estimator (Michalet and Berglund, 2012) that was adapted to incorporate variable localization precision and will be described in detail elsewhere.

### Instantaneous Velocity Estimation

The instantaneous velocity was estimated with a window-based linear approximation scheme. A sliding window size of seven localizations around each trajectory localization was fit to a linear model and the velocity of that linear segment was used to approximate the true instantaneous velocity of the trajectory at that instant. This method smooths over the noise present in individual localizations but still captures small time-scale changes in particle trajectory.

## Results

### Characterization of Brain-Derived Neurotrophic Factor Retrograde Transport by Single Particle Brain-Derived Neurotrophic Factor-Quantum Dot Quantification

Spatiotemporal trafficking dynamics of BDNF in central nervous systems neurons has previously been studied using microfluidic (Poon et al., 2011; Zhao et al., 2014; Wang et al., 2016; Fang et al., 2017) devices, which allow spatial guidance and fluidic isolation of distal axons from cell bodies, but the same knowledge in sensory nervous system neurons is lagging. The microfluidic devices used in this study consisted of a cell body compartment connected to an axonal compartment via a series of microfluidic channels (450 μm in length, 5 μm in width) (Figure 1A). This experimental paradigm allowed us to observe retrograde BDNF-QD axonal trafficking at designated time points following BDNF-QD stimulation of the sensory axons in the axonal compartment. Using our customized software algorithm (see section “Materials and Methods”), we detected and tabulated single BDNF-QDs in fixed DRG neuronal cultures with high specificity, sensitivity, as well as high precision spatial localization (Figure 1B). The combination of these techniques enabled us account for discrete BDNF-QDs undergoing retrograde transport, allowing us to obtain more accurate information compared to averaged information obtained from imaging general fluorescence levels.

**FIGURE 2:**
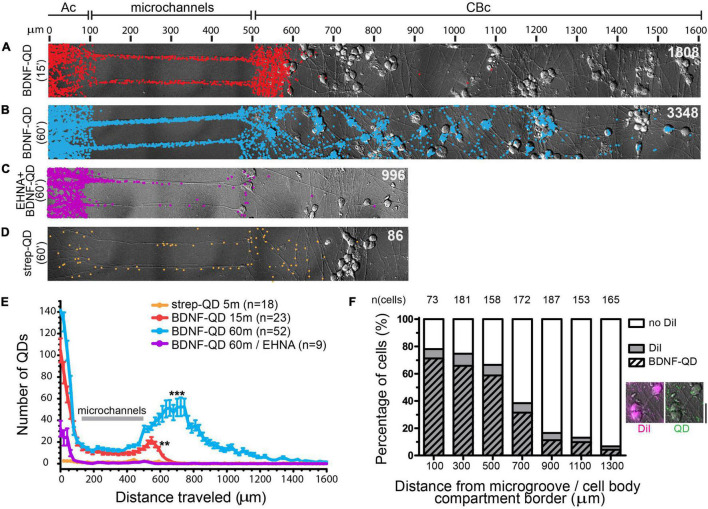
Dynein-mediated retrograde transport of BDNF-QD complexes along axons in DRG sensory neurons by single-molecule quantification. **(A–D)** Images representing overlays of DIC and spatial distribution of QDs (colored dots) following BDNF-QD stimulations. **(A)** Distribution of BDNF-QDs following a 15 min BDNF-QD stimulation (red dots). **(B)** Distribution of BDNF-QDs following a 60 min BDNF-QD incubation (blue dots). **(C)** Distribution of BDNF-QDs following a 60 min BDNF-QD incubation (purple dots) in the presence of 1 μm EHNA, a dynein blocker. **(D)** Distribution of streptavidin (strep)-QDs following a 60 min strep-QD incubation (orange dots). All images are overlays of DIC and Matlab-generated QD dots; total numbers of QDs are indicated in white (top right in each image); scale (μm) is shown at the top of panel **(A)**. Axonal compartment (Ac), Cell Body compartment (CBc). **(E)** Quantitative summary graph showing the QD spread over time for the conditions described in panels **(A–D)** (*y*: frequency, *x*: distance traveled (bins of 200 μm) from point of addition). Results are presented as the average number of QDs detected/bin ± S.E.M. Gray line indicates the microchannels position; *n* = number of cultures analyzed; one-way ANOVA (*p* < 0.0001) with Dunnet’s multiple comparison test (***p < 0.0001, **p < 0.001 compared to St = Q control). **(F)** Correlation of strong DiI labeling and a high number of BDNF-QDs (paired *t*-test, *p* = 0.0006). Each bar represents percentage of cell bodies found inside 200 × 200 μm areas moving away from the microchannels (distance = 0 μm starts with cell body compartment, it does not include microchannels), showing absence or presence of DiI in the cell bodies (white and gray, respectively), and the percentage of DiI and BDNF-QD positive cells (gray with stripes). Examples of cell bodies labeled with DiI (magenta) and BDNF-QDs (green) are shown to the right of the graph, images were taken at 100 μm from the microchannel/cell body edge (scale bar = 25 μm).

We first sought to determine and quantify the time course of BDNF-QD retrograde trafficking in DRG sensory neurons grown in microfluidic devices. Stimulation of the axonal compartment with BDNF-QD for 15 min resulted in the translocation of BDNF-QD to axons both in microchannels and in the first 100 μm of the cell body compartment (Figures 2A,E). A longer BDNF-QD stimulation (60 min) resulted in a 2-fold increase in the number of BDNF-QDs in axons in the microchannel and cell body compartment, and the translocation of BDNF-QD to cell bodies up to 1,000 μm away from the microchannels (Figures 2B,E). As expected, the cell body chamber containing BDNF-QD labeled cell bodies was characterized as a gradient, with the greatest number of labeled cell bodies near the microchannel openings. To determine if the translocation of BDNF-QD from the axonal compartment to the cell body compartment is microtubule-dependent, we measured retrograde transport of BDNF-QD in the presence of 1 μM EHNA, a broad spectrum inhibitor of the retrograde motor dynein (Yano et al., 2001). Treatment of the axonal compartment with EHNA prevented translocation of BDNF-QD from the axonal compartment to the cell body compartment (Figures 2C,E) similarly to previous studies (Yamada et al., 2008; Terenzio et al., 2014; Cristofani et al., 2017; Escudero et al., 2019). This supports the notion that translocation of BDNF-QD from the axonal compartment to the cell body compartment is mediated by dynein and not due to free diffusion through the microchannels. This is consistent with previous studies examining the retrograde trafficking of radiolabeled NGF (Ye et al., 2003) and Trk NT receptors (Yano et al., 2001). In addition, the BDNF-QD counts were significantly above the negative control (streptavidin-QD), indicating that non-specific binding of BDNF-QDs to neurons was rare (Figure 2D). As an additional control experiment, we used DiI, a lipohilic neuronal tracer that was added in conjunction with BDNF-QDs, to show a high degree of BDNF-QD and DiI colocalization in cell bodies, indicating that the cell bodies that contained BDNF-QDs are the ones with axons in the axonal compartment (Figure 2F). Taken together, these results demonstrate that BDNF-QD is translocated from axons to the cell body in a dynein-dependent manner, and that our single particle counting method provides a highly sensitive measure of quantifying BDNF-QD retrograde transport with high spatial resolution.

### Brain-Derived Neurotrophic Factor-Quantum Dot Axonal Retrograde Transport in Live Neurons Is Comprised of Diverse Molecular Motions That Vary Over Time and Distance

**FIGURE 3:**
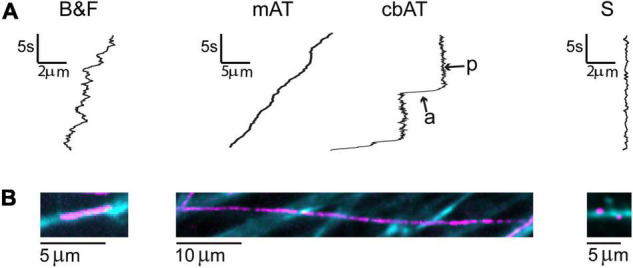
Classes of BDNF-QD motions observed during retrograde transport in DRG neurons. **(A)** General schematics of the types of motion: explorative-like back-and-forth motion seen in axons in the axonal compartment (B&F), where BDNF-QDs were added; active transport interspersed with pauses along the axons in the microchannels (mAT) and cell body compartment (cbAT) showing active (a) and paused (p) phases; and stationary-like (S). Scale: t = time (s), d = distance (μm). **(B)** Max projection of BDNF-QD motion (video, magenta) superimposed on WGA-488 images of neurons (cyan blue).

**FIGURE 4:**
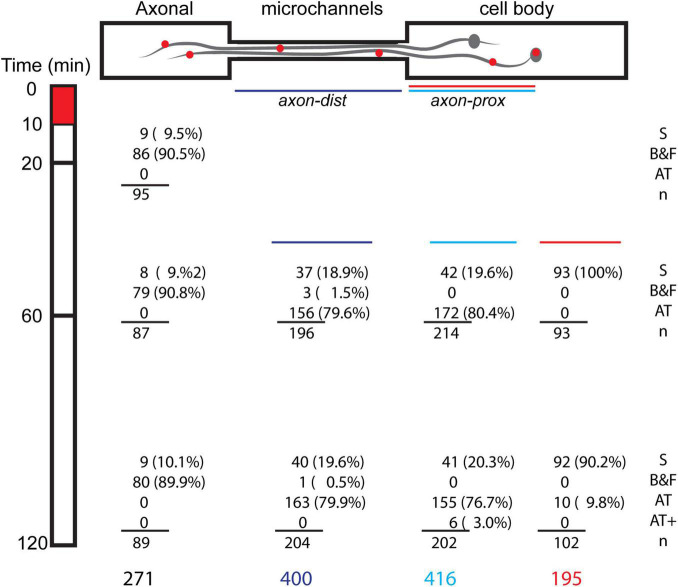
Spatial temporal distribution of classes of BDNF motions. DRG neurons were stimulated for 10 min with 250 pM BDNF-QD added to the axonal tips (*t* = 0–10 min, red box), and then imaged along the axonal compartment (Ac), microchannel (mCh) and cell body compartment (CBc) at different time periods (*t* = 10–120 min). Numbers (*n*) and percentages (%) represent the BDNF-QD counts for each position in time (time point/distance in microchannel), and are classified by type of motion: stationary (S), back-and-forth motion (B&F), active transport (AT) and active transport of BDNF-QD pairs (AT+).

To study the dynamics of retrograde trafficking, we used live cell imaging of discrete transported BDNF-QD molecules. Qualitative trajectories obtained from twenty-five independent DRG sensory neuronal cultures showed great heterogeneity in the BDNF-QD motions imaged in the axonal compartment, microchannels and cell body compartment at various time points (between 10 and 120 min post-BDNF-QD treatment). During our axonal observations, we were able to detect BDNF-QD motions on axons that fell into three general categories: (1) back-and-forth dynamics (B&F, Figure 3, left), (2) rapid linearly-directed motion that was interspersed with pauses and classified as active transport (AT, Figure 3, middle), and (3) stationary with vibration-like dynamics (S, Figure 3, right). A few BDNF-QDs and all control QDs were detected directly sitting on the cover glass were completely static and not taken into account for future analysis. The frequency of each type of these motions changed over time and was dependent on the location within the compartment (axonal endings, axons and cell bodies) (Figure 4). Further quantitative physical measurements corresponding to each of these classes of motions revealed underlying biophysical features that were not apparent in qualitative observations and are described in subsequent sections, with special attention to the rapid active transport.

In the axonal compartment, where the BDNF-QDs were added, the B&F diffusive-like motion was prevalent along short stretches of the axons, and remained so throughout the 2-h duration of the experiment (Supplementary Video 1 and Supplementary Figure 1). This B&F motion was characterized by a linear, one-dimensional motion that repeatedly changed directionality yet remained confined to a narrow elongated domain (3–7 μm in length). In addition to the B&F motion, a small number of BDNF-QDs on the axons (<10%) in the axonal compartment appeared to be vibrating in place and were termed “stationary.”

In the microchannel compartment, which contained long stretches of axons (150 μm), BDNF-QDs exhibited both stationary and active transport motions. Active transport was the predominant motion (*n* = 319/400) and was composed of fast and linear, active segments interspersed with paused phases (Supplementary Video 2). The number of BDNF-QDs undergoing active transport across the microchannels increased over time, doubling between 20 and 60 min, and then remained constant thereafter (60–120 min). In almost all instances, active transport was directed toward the cell bodies, with only two cases where we observed BDNF-QDs that showed a temporary reversal in direction. In addition to the active transport, a very small proportion of BDNF-QDs exhibited B&F motion (*n* = 4/400), and this was observed in the axons that were near the axonal compartment, where this motion was common. Some BDNF-QDs also appeared to be stationary (stationary, *n* = 77/400) but then resumed active transport at a later point in time.

In cell body compartment, BDNF-QDs traveling along long stretches of axons (175 μm) experienced active transport after 30 min post-BDNF-QD stimulation (*t* = 30–120 min). This transport was comprised of fast linear active segments interspersed with paused phases (*n* = 327/416, Supplementary Video 3), similar to that observed in the microchannels. Interestingly, at later times (*t* = 90–120 min; *n* = 6/202), we found BDNF-QDs moving by active transport along the same axon in closely paired (<1 μm) configurations. Inside the cell bodies, the BDNF-QDs were predominantly classified as “stationary” (*n* = 185/195); however, some BDNF-QDs (10/195) underwent active transport during later time points (*t* = 90–120 min) that and was characterized by fast curvilinear motions composed of active and paused phases (Supplementary Video 4 and Supplementary Figure 2).

Taken together, these data indicate that retrograde trafficking of BDNF-QD following stimulation of axonal endings comprised of a complex s mixture of heterogeneous motions. Classification of these fine molecular motions show that they varied not only as a function of the neuronal compartment (B&F in the axonal compartment, active retrograde transport in the axons, mainly lack of motion inside the cell bodies), but also varied over time (active transport of single vs pairs of BDNF-QDs).

### Detailed Kinetics of Brain-Derived Neurotrophic Factor Retrograde Transport by Live Single Particle Tracking

To obtain deeper insight into the nature of the movement of BDNF-QD along the sensory neuronal axons, we performed quantitative trajectory analysis all active transport BNDF-QD motions. We sought to capture the detailed dynamics of by tracking and computing several different parameters shown in Table 1. The features of QD brightness and photostability allowed us to obtain longer videos at higher frames rate (60 ms) than previously reported, while maintaining high resolution (e.g., effective pixel size of 245 nm) (Supplementary Video 5). This allowed us to resolve both the short- and long-timescale processes of single BDNF-QD trafficking along axons.

**TABLE 1:** Quantitative parameters used in trajectory analysis of each class of brain-derived neurotrophic factor-quantum dot (BDNF-QD) motion.

Parameter	Symbol	Units	Description	Equation
Net displacement	*d*_net_	μm	Net distance traveled from beginning to end	*d*_net_=||*p*_*N*_−*p*_1_||
Max displacement	*d*_*max*_	μm	Maximum displacement observed between any two positions	*d*_max_=max*i*,*j*||**p**_*i*_−**p**_*j*_||
Net speed	*s*_*net*_	μm/s	Net distance divided by total time	$s_{\text{net}}=\frac{d_{\text{net}}}{\left(t_{N}-t_{1}\right)}$
Mean curvilinear speed	$\bar{s}$	μm/s	Mean of instantaneous speed over each displacement	$\bar{s}=\frac{1}{N-1}\,\sum_{i=1}^{N-1}\frac{||p_{i+1}-p_{i}||}{t_{i+1}-t_{i}}$
Linearity ratio (Linearity of forward progression)	*r*_*lin*_	–	Ratio of net speed to mean speed. Values close to 1 indicate linear motion. Values close to 0 indicate random motion	$r_{\text{lin}}=\frac{s_{\text{net}}}{\bar{s}}$
Diffusion constant	*D*	μm^2^/s	Maximum likelihood estimate of diffusion constant	See “Materials and Methods”

*A trajectory with N localizations is defined by a sequence ${\left\{p_{i}\right\}}_{i=1}^{N}$, of measured 2D particle positions and sequence ${\left\{t_{i}\right\}}_{i=1}^{N}$ (Zhang et al., 2010) of measurement times. Given this definition we define several trajectory statistics that are commonly used in characterizing particle motion (Meijering et al., 2012).*

### Retrograde Brain-Derived Neurotrophic Factor-Quantum Dot Active Transport Is Faster in Axons That Are Proximal to the Cell Bodies Due to Shorter Pauses

**FIGURE 5:**
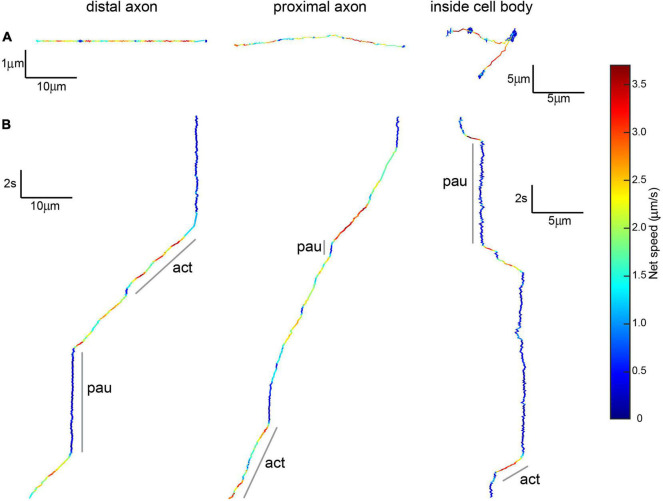
Brain-derived neurotrophic factor-quantum dots undergoing active transport show active and paused phases. **(A)** Top view of 20 s-long BDNF-QD track showing active transport along distal and proximal axons and inside cell bodies, colors show instantaneous speed estimates. Scale: x = distance (10 μm for axonal, 5 μm for inside the cell body), y = distance (1 μm for axonal, 5 μm for inside the cell body). **(B)** X-views of the same BDNF-QD tracks shown in panel **(A)**, pointing out active and paused phases (a, p). Scale: y = time (2 s), x = distance (10 μm for axonal, 5 μm for inside the cell body).

**FIGURE 6:**
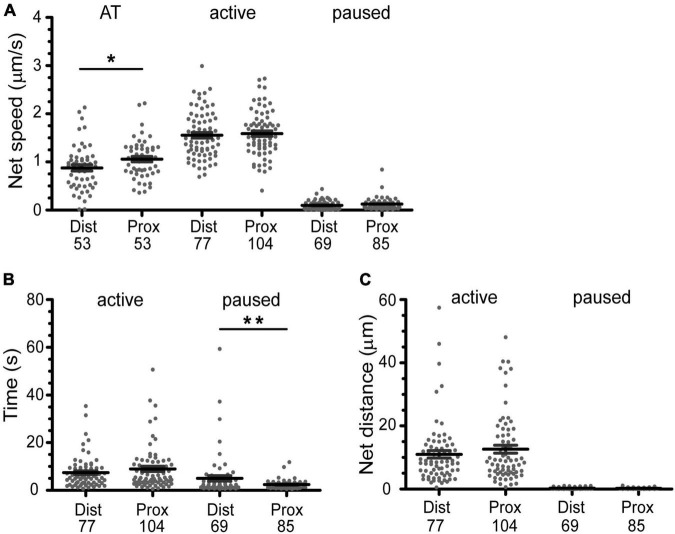
Active transport of BDNF-QDs is faster in axons proximal to cell bodies due to shorter pause phases. **(A)** The overall active transport (AT) was faster for BDNF-QDs proximal to the cell bodies; unpaired *t*-test, *p* = 0.0354 (AP), 0.8434 (A), 0.0671 (P). **(B)** The time duration of the paused phases is on average shorter for BDNF-QDs proximal to the cell bodies; unpaired *t*-test, *p* = 0.6121 (A), 0.0022 (P). **(C)** The BDNF-QD net displacement during the active phases was not significantly different between proximal and distal axons, the net displacement for the paused phases was not either; unpaired *t*-test, *p* = 0.6419 (active), 0.0780 (paused). Box plots show the distribution of the values for discrete BDNF-QDs, as well as the mean ± SEM. *X-axis* indicates position of the BDNF-QDs in the axons: distal (Dist, microchannels) and proximal (Prox, cell body compartment). *p < 0.05; **p < 0.01. *n* is shown at the bottom of each graph (below Dist and Prox).

Our qualitative observations indicated that linear active transport was the predominant motion in axons found in the microchannel (distal) and cell body (proximal) compartments (Table 1 and Figure 5). The average net speed (S_*net*_) of BDNF-QD active transport in axons was 1.0 ± 0.4 μm/s. However, when we compared the net speed between distal and proximal axons (defined as axons in the microchannels and axons in the cell body chamber, respectively, Figure 4), we found that BDNF-QDs were transported faster in the proximal axons compared to the distal axons (Figure 6A; 1.058 ± 0.403 and 0.876 ± 0.475 μm/s, respectively, *n* = 53 for each group). Another way to look at active transport is the linearity of the forward progression (*r*_lin_), which gives a relative measure of the tendency of a trajectory to move in a linear and directional manner. This value was also significantly greater in the proximal axons compared to the distal axons (0.469 ± 0.197 and 0.619 ± 0.172, respectively, *n* = 53 each, unpaired *t*-test *p* < 0.0001), which corresponds to more efficient transport in the proximal axons.

These observations show that transport is overall faster and more efficient closer to the cell body, but both *s*_*net*_ and *r*_*lin*_ are summary statistics that describe the trajectory as a whole. Our high-spatio temporal resolution images show that the net motion of the actively transported BDNF-QDs is actually composed of periods of linear, directed transport interspersed with pauses where the QD remains confined to the track, until it resumes motion. To better understand how the active transport behavior differed with distance to the cell body, we compared the net speed of the active motion between pauses in the trajectories from proximal and distal axons. For this comparison, we included the active phases with known beginning and end points (i.e., between two paused phases). Interestingly, the net speed of the active phases was not significantly different between proximal and distal axonal regions (Figure 6A; 1.544 ± 0.446 and 1.531 ± 0.486 μm/s, respectively; *n* = 104 and 77). Moreover, a comparative analysis of the duration of the active phases between proximal and distal axons also showed that they were not significantly different (Figure 6B, 6.721 ± 6.634 and 7.218 ± 6.345 s, respectively; *n* = 104 and 77). This indicated that the overall faster active transport near the proximal axons was not due to faster net speed or longer active phases in this region compared to the distal axons.

While the characteristics of the active phases of the active/paused transport behavior appear to be identical for proximal and distal parts of the axon, an analysis of the duration of the paused events was on average significantly shorter in the proximal versus the distal axons (Figure 6B, 1.931 ± 1.842 and 5.285 ± 9.420 s, respectively; *n* = 69 and 85). In addition, the number of BDNF-QDs that paused for less than 5 s in the axons proximal to the cell bodies was higher than for those in the distal axons (95 and 76.8%, respectively). To eliminate the possibility of a reduced frequency of paused phases contributing as well to the faster proximal active transport, we compared the net displacement of the active phases in proximal and distal axons, reasoning that these displacements would be larger when the number of pauses is reduced. Our results showed that there is no significant difference in the net displacement of active phases between proximal and distal axons (Figure 6C, 9.890 ± 9.385 and 10.56 ± 9.741 μm, respectively; *n* = 104 and 77). Taken together, our results showed that the faster active transport of BDNF-QDs in axons proximal to the cell bodies compared to BDNF-QDs in distal axons was due to significantly shorter durations for the paused phases, instead of differences in the net speed or duration of the active phases.

### The Brain-Derived Neurotrophic Factor-Quantum Dot Active Transport Inside Cell Bodies Is Slower Than in Axons

Active transport was also detected in trajectories found inside the cell bodies (*n* = 9/102). Even though no BDNF-QD molecules were directly observed entering the cell body, the use of the microfluidic chamber implies that these BDNF-QDs must have entered the cell body via axonal intracellular transport. The maximum 2D (x,y) displacement of the trajectories inside the cell bodies, ranged between 2.334 and 8.037 μm with an average of 3.259 ± 5.295 μm. As in axons, active transport inside cell bodies was characterized by active phases interspersed with paused phases, with active phases showing directed curvilinear motion (Figures 5A,B-inside cell body). We compared the net speed (*s*_net_) and duration of active (*n* = 20) and paused phases (*n* = 17) inside cell bodies to the axonal active and paused phases (previous section). Our results showed: (1) the net speed for the active phase inside the cell bodies was significantly slower than in the distal and proximal axons (1.10 ± 0.55 vs 1.5 μm/s in axons; 1-way ANOVA *p* = 0.0006, Bonferroni’s post-hoc test), (2) the time duration of the active phase was significantly shorter than in axons (1.955 ± 1.945 vs 6.932 ± 6.500 s, 1-way ANOVA *p* = 0.0032, Bonferroni’s post-hoc test), and (3) that the average duration of the paused phases inside cell bodies was more similar to the pauses in distal axons than in proximal ones (4.953 ± 3.777 s compared to 5.285 ± 9.420 and 1.931 ± 1.842 s, respectively). We conclude that the average net speed of BDNF-QDs during active transport inside cell bodies was slower than in axons, with shorter active and longer paused phases (a summary of axonal and cell body speed, active and pause durations is shown in Table 2).

**TABLE 2:** Comparative table showing active speed, as well as active and paused durations of BDNF-QDs in distal axons, proximal axons, and inside cell bodies.

	Distal axon	*n*	Proximal axon	*n*	Cell body	*n*
Active speed (μm/s)	1.531 ± 0.486	77	1.544 ± 0.446	104	1.1 ± 0.55	20
Active phase (s)	7.218 ± 6.346	77	6.721 ± 6.634	104	1.9955 ± 6.932	20
Paused phase (s)	5.288 ± 9.420	69	1.931 ± 1.842	85	4.953 ± 3.777	17

*Active phase is defined as the duration between two pauses, and paused phase as the duration between two active phases. Values indicate mean ± SEM, and the total number (n) analyzed.*

### Coordinated Active Transport of Multiple Brain-Derived Neurotrophic Factor-Quantum Dots

**FIGURE 7:**
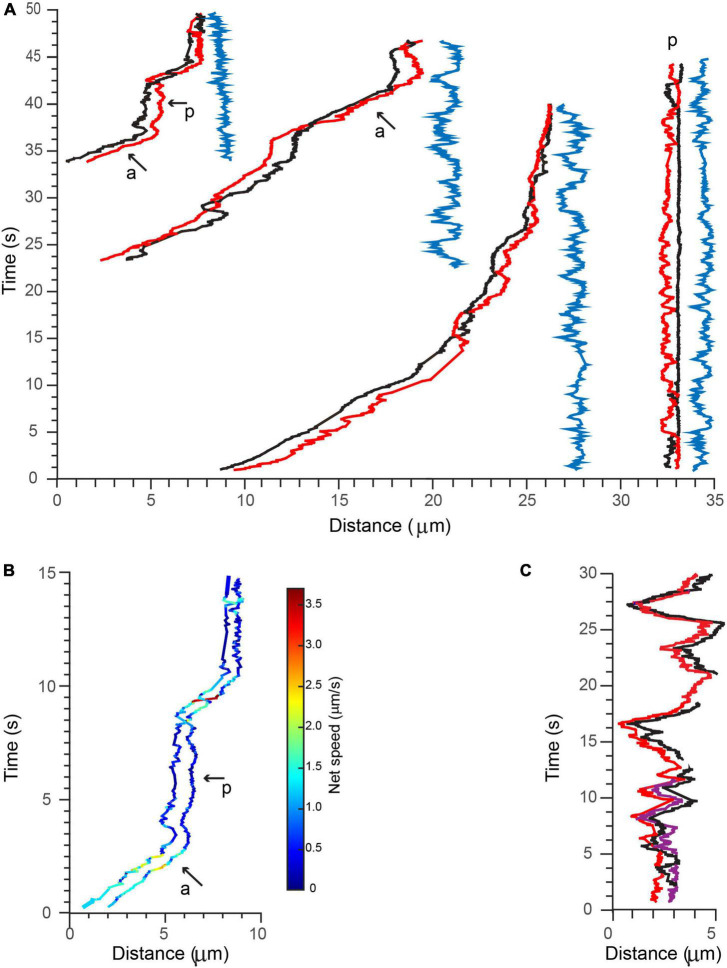
Active transport of multiple BDNF-QDs. **(A)** Three examples of BDNF-QDs traveling as coordinated pairs (red and black lines) during active transport in proximal axons (three most left side), and one example during a paused phase (right side). The distance between the (red and black) QDs was dynamic and never exceeded 2 μm, and it is shown on the right of each paired trajectory (blue lines). Active (a) and paused (p) phases are also indicated for the most left example. **(B)** The net speed of each BDNF-QD in the pair during the active and paused phases is similar. Trajectories are color-coded for speed (color bar on right). **(C)** Example of a BDNF-QD triplet during active transport in proximal axons. Image is color-coded for discrete QDs (red, black, and purple).

Following 90 min after initial BDNF-QD treatment, we observed the surprising occurrence of BDNF-QDs that traveled in coordinated pairs and even triplets, along a single axon (Figures 7A,C; Supplementary Video 6, *n* = 6). By visual inspection, the motion of these BDNF-QDs seemed loosely coupled; however, trajectory analysis indicated that the transport of these multiple BDNF-QDs was highly coordinated, and that distances between two BDNF-QDs were always less than 2 μm (Figure 7A, blue traces). It is unclear whether these instances of multiple BDNF-QDs were composed of discrete BDNF-QDs traveling in the same endosomal compartment or whether BDNF-QDs were composed of two different endosomes in sequence since we were not able to detect the fusion of two BDNF-QDs at earlier time points. However, the possibility that BDNF-QD were traveling in the same endosome compartments is likely, since transport was highly coordinated, always a short distance between two BDNF-QDs (never more than 2 μm), and maintained for long durations (40 s) and over significant distances of more than 50 μm (Supplementary Video 6). We also observed one BDNF-QD pair in there one BDNF-QD was stationary whereas the other BDNF-QD appeared to either rotate or move toward and away from the other. Moreover, the net speed, net displacement, and duration of the active and paused phases of active transport were the same for both BDNF-QDs in a pair (Figure 7B). Similar coordinated inter-distances were observed for an endosome containing three BDNF-QDs that was seen actively transported (Figure 7C).

## Discussion and Conclusion

Using a combination of microfluidic devices for cell culture along with high-resolution single particle QD tracking, we conducted a systematic study to characterize the time resolved, molecular motions of discrete BDNF-QD endosomes undergoing long-range retrograde transport (hundreds of microns) within the axon of DRG sensory neurons. The first key finding is that a complex, heterogeneous mixture of sub-micron motions underlies prior macroscopic observations of axonal BDNF retrograde transport. These molecular motions are distinct and are not exclusive to active motor-driven transport (active speed) but also include the dynamics and durations of phases of non-motor driven transport (pauses) which contribute to overall net transport speed. While non-motor driven transport has been observed, its prevalence during long-range axonal transport and the impact of its contribution to net BDNF-QD retrograde speed has not been previously recognized. This finding underscores the importance of factors other than active motor transport in governing retrograde axonal transport. The second key finding is that these heterogeneous but distinct molecular-scale dynamics are organized by cellular regions: distal axons, along the axon shaft, and in approach to the cell soma. The new observation of these spatially distinct molecular scale dynamics points to the subcellular axonal architecture along with its molecular components within axons as regionally heterogeneous and contributing to shaping overall retrograde transport speed.

We find many examples illustrating the heterogenous composition of molecular-scale motions that include but are not exclusive to motor transport. Such non-motor and motor-driven transport dynamic, when heterogenous, are distinct and organized by cellular regions and both shape the net motion of BDNF retrograde transport along axons (Figure 4 and Table 2). At the distal tips of axons, BDNF-QD motion is diffusive-like, as characterized by frequent changes in direction and small net displacements, wherein initial BDNF-QD diffusive motion has not yet initiated active transport. This behavior is similar to diffusive behavior observed in hippocampal and cortical neuronal systems (Cui et al., 2007). Along axons, the active transport components of overall BDNF retrograde transport are mediated in part by dynein motors (Figure 2C). As characteristic of dynein motors, active transport in DRG neural axons rarely showed directional reversal and was blocked by EHNA. The speed of active transport was 1.0 ± 0.4 μm/s; this is consistent with previously published data for NT transport (Cui et al., 2007; Sung et al., 2011; Xie et al., 2012; Zhao et al., 2014). Notably, these motors do not solely dictate overall BDNF retrograde transport speed. At axons near the cell bodies, we found that BDNF retrograde transport is faster than at distal axons, interestingly, this overall speed is not produced entirely by faster or longer phases active transport but is shaped by shorter pauses between phases of active transport (average of 1.9 vs 4.7 s; distal vs proximal axons, Figure 6 and Table 2). Variation in BDNF retrograde transport was also observed at the cell body, wherein motion in the cell body was slower compared to transport along the axonal shaft. This slower BNDF transport in the cell body was the product of shorter and slower active transport phases compared active transport in axons (Figure 6 and Table 2).

What factors shape the molecular dynamic features that we observe and contribute to shaping BDNF axonal transport and signaling? Our results showing dependence of spatial location suggest that ultrastructural features in axons may produce changes in molecular dynamics that contribute to overall NT transport. Such differences in subcellular architecture along the axons and within the cell body could in turn regulate the chemical binding properties of different molecular motors such as the availability of microtubule-associated accessory proteins and post-translational modifications (PTMs) (Hammond et al., 2008; Franker and Hoogenraad, 2013; Sirajuddin et al., 2014; Song and Brady, 2015). For example, it has been proposed that microtubule stability varies along the axonal length, with the distal polymer end turning over more rapidly than elsewhere in the axon (Black et al., 1996). Experimental studies that will require molecular-genetic manipulation of specific PTMs and their influence on microtubule length will need to be done. Could downstream activation in turn shape BDNF-endosomal dynamics we observed? Prior studies have shown that early gene expression (mRNA) and their corresponding protein products (e.g., c-Fos, Arc, and pCREB) are induced at >1–2 h following BDNF and/or NGF treatment to cultured neurons (Riccio et al., 1997; El-Sayed et al., 2011). Moreover, while localized application of BDNF to dendritic cortical neurons produced c-Fos and Arc protein, localized application of BDNF to axonal terminal did not provoke protein production (Cohen et al., 2011). These studies suggest that downstream signaling is unlikely a factor in further shaping BDNF-trafficking dynamics. Finally, it will be valuable to perform ultrastructural studies of axons to look at the role of features such microtubule lengths and molecular crowding features along the axons and in cell bodies in shaping BDNF trafficking dynamics. Further work in this realm would increase understanding of factors governing signaling in defective in axonal trafficking, as in the case of ultrastructural changes that are altered in neurodegenerative diseases (Zuccato and Cattaneo, 2009; Millecamps and Julien, 2013).

## Data Availability Statement

The raw data supporting the conclusions of this article will be made available by the authors, without undue reservation.

## Ethics Statement

The animal study was reviewed and approved by Institutional Animal Care and Use Committee of the Oregon Health and Science University (Protocol Number: IS00001990).

## Author Contributions

AV-S performed the experiments. AV-S, MO, and DR-J analyzed the data. MC provided DRG culture expertise. KL and TV provided high-resolution tracking expertise. AV-S and TV wrote the manuscript with input from all authors. All authors approved the submitted version of the manuscript.

## Conflict of Interest

The authors declare that the research was conducted in the absence of any commercial or financial relationships that could be construed as a potential conflict of interest.

## Publisher’s Note

All claims expressed in this article are solely those of the authors and do not necessarily represent those of their affiliated organizations, or those of the publisher, the editors and the reviewers. Any product that may be evaluated in this article, or claim that may be made by its manufacturer, is not guaranteed or endorsed by the publisher.

## Footnotes

1
www.fiji.sc
