# Supplementary material for: Molecular-Scale Dynamics of Long Range Retrograde Brain-Derived Neurotrophic Factor Transport Shaped by Cellular Spatial Context
Source: Front Neurosci. 2022 Mar 31;16:835815. doi: 10.3389/fnins.2022.835815 (PMC9008462; doi:10.3389/fnins.2022.835815)
Supplement: Supplementary file 1 [file Data_Sheet_1.docx]

**SUPPORTING INFORMATION**

**sFigure1:** This figure shows a reference for the BDNF-QD trajectory shown in sVideo1 relative to axons in the axonal compartment. The image is a maximum projection of the BDNF-QD video (magenta) overlaid onto an image that shows the neurons labeled with WGA488 (cyan blue). White box shows area of the video, white arrow indicates the trajectory shown in sVideo1. Scale bar = 10 µm.

**sFigure2:** This figure shows a reference for the BDNF-QD trajectory shown in sVideo4 relative to the cell body membrane. The image is a maximum projection of the BDNF-QD video (magenta) overlaid onto an image that shows the neurons labeled with WGA488 (cyan blue). White box shows area of the video shown in sVideo1. Scale bar = 10 µm.

**sVideo 1:** This video shows a single particle trajectory exhibiting the explorative diffusive (aka. back-and-forth) motion of a BDNF-QD that is typical of the initial QD activity in the axonal compartment, where BDNF-QDs were added. The trajectory is confined to the axon and explores small side branches. The movie shows dynamic tracking from a 2D perspective as well as a 3D flyover of a static rendering of the trajectory in x, y, and time dimensions. See also sFigure1 for a reference of the BDNF-QD trajectory relative to axons.

**sVideo 2:** This video shows tracking of several BDNF-QDs moving along distal axons within the microchannel. Most of the selected trajectories exhibit the active/pause transport dynamics that characterizes the primary motion observed in axons along the microchannel. Some trajectories are apparently stationary or confined and are shown for comparison to the active behavior. The movie shows dynamic tracking from a 2D perspective where colors distinguish different particle trajectories, and we also show a 3D flyover of a static rendering of the trajectories in x, y, and time dimensions. Of particular interest is the nature of the pauses in the active transport trajectories which are well resolved at our high spatio-temporal tracking resolutions.

**sVideo 3:** This video shows tracking of several BDNF-QDs moving along proximal axons in the cell body compartment. At the end of the movie, the top view gets turned into a side view showing the trajectory being rotated around its time axis to show 3D motions. The pauses in the active/paused transport dynamics are also evident in this movie. Of note, these pauses are shorter than those experienced in active transport trajectories in the more distal regions of the axons in the microchannel well as shown in sVideo2.

**sVideo 4:** This video shows tracking of several BDNF-QDs moving inside a cell body. The trajectories show a dynamic switch between active transport along curvi-linear structures to more stationary confined diffusive behavior. Also of note is the apparent interaction of two of the BDNF-QD complexes. Because of the inability to individually distinguish overlapping and interacting QDs the trajectories are divided at the beginning and end of interaction time-points. These trajectories divisions are distinguished visually by color of the trajectory. See also sFigure2 for a reference of the BDNF-QD trajectory relative to cell body.

**sVideo 5:** This movie shows a BDNF-QD moving by active/paused transport dynamics in a proximal portion of the axon within the cell-body well. The video is rotated from a 2D to a 3D projection showing the time component of the trajectory as it is being tracked. We also zoom in on this trajectory at several different scales to show the spatio-temporal details that can be resolved with our single particle tracking procedure. On frames with an accepted localization a blurry disk is drawn to show the 95% confidence bounds on the true position of the particle in that frame.

**sVideo 6:** This movie shows the trajectories for two nearby BDNF-QDs both moving by active/paused transport over the same axon. These QDs were observed in a proximal portion of the axon in the cell-body well. Note that both QDs move in the same direction and remain within <2um of each other throughout the tracked portion. They both experience periods of active motion and pauses, but these events are not necessarily coordinated between the two QDs. It should also be noted that when the QDs move sufficiently close to each other that their diffraction-limited spots overlap, it becomes difficult to individually resolve their location. For this reason, it is possible the identities of the two particles switch after one of these adjacency events. Still, we can report the relative displacement between the QDs even if the true identities cannot be confirmed after a potential interaction.
