# Supplementary figures and images for: Molecular-Scale Dynamics of Long Range Retrograde Brain-Derived Neurotrophic Factor Transport Shaped by Cellular Spatial Context
Source: Front Neurosci. 2022 Mar 31;16:835815. doi: 10.3389/fnins.2022.835815 (PMC9008462; doi:10.3389/fnins.2022.835815)

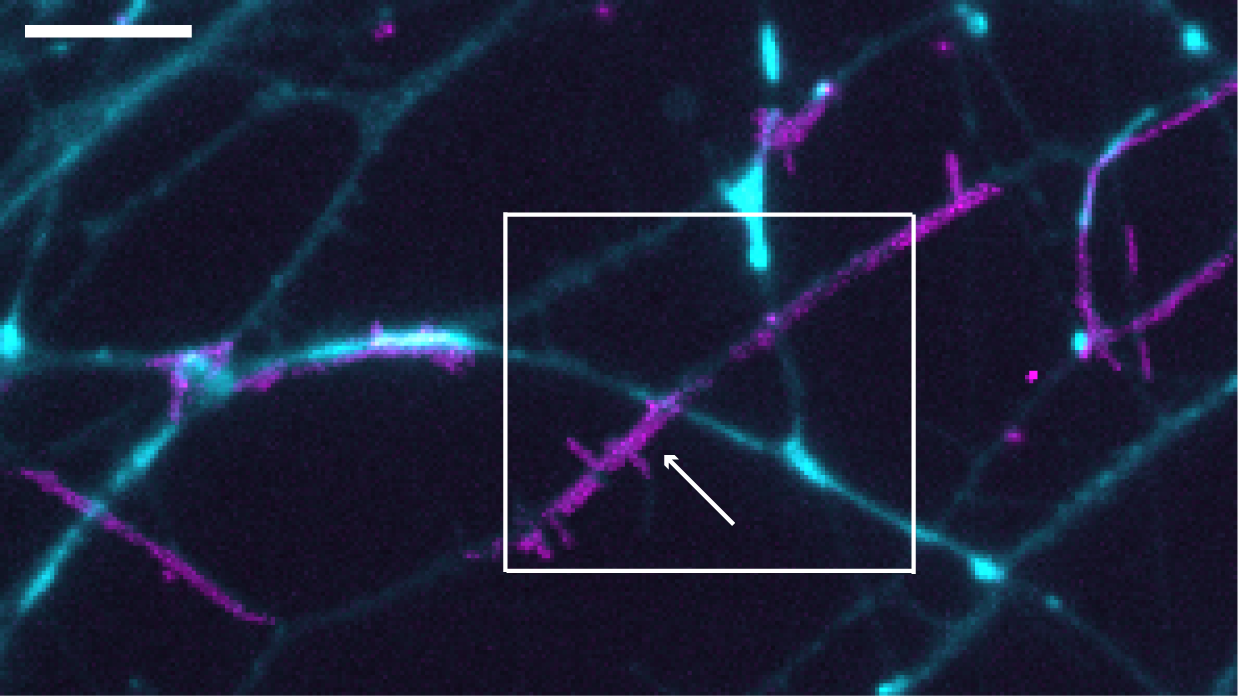

Supplement: Supplementary file 2 [file Image_1.tif]

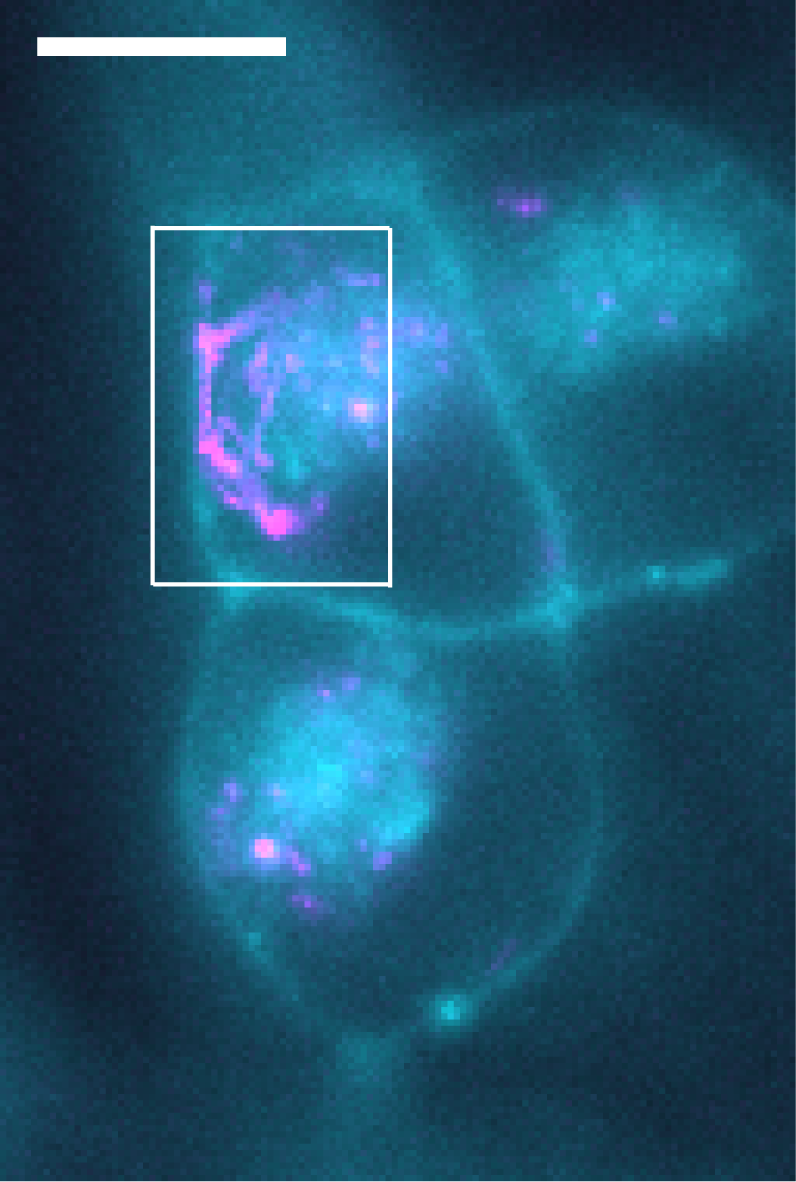

Supplement: Supplementary file 3 [file Image_2.tif]
